# Supplementary figures and images for: Predicting the target landscape of kinase inhibitors using 3D convolutional neural networks
Source: PLoS Comput Biol. 2023 Sep 5;19(9):e1011301. doi: 10.1371/journal.pcbi.1011301 (PMC10508635; doi:10.1371/journal.pcbi.1011301)

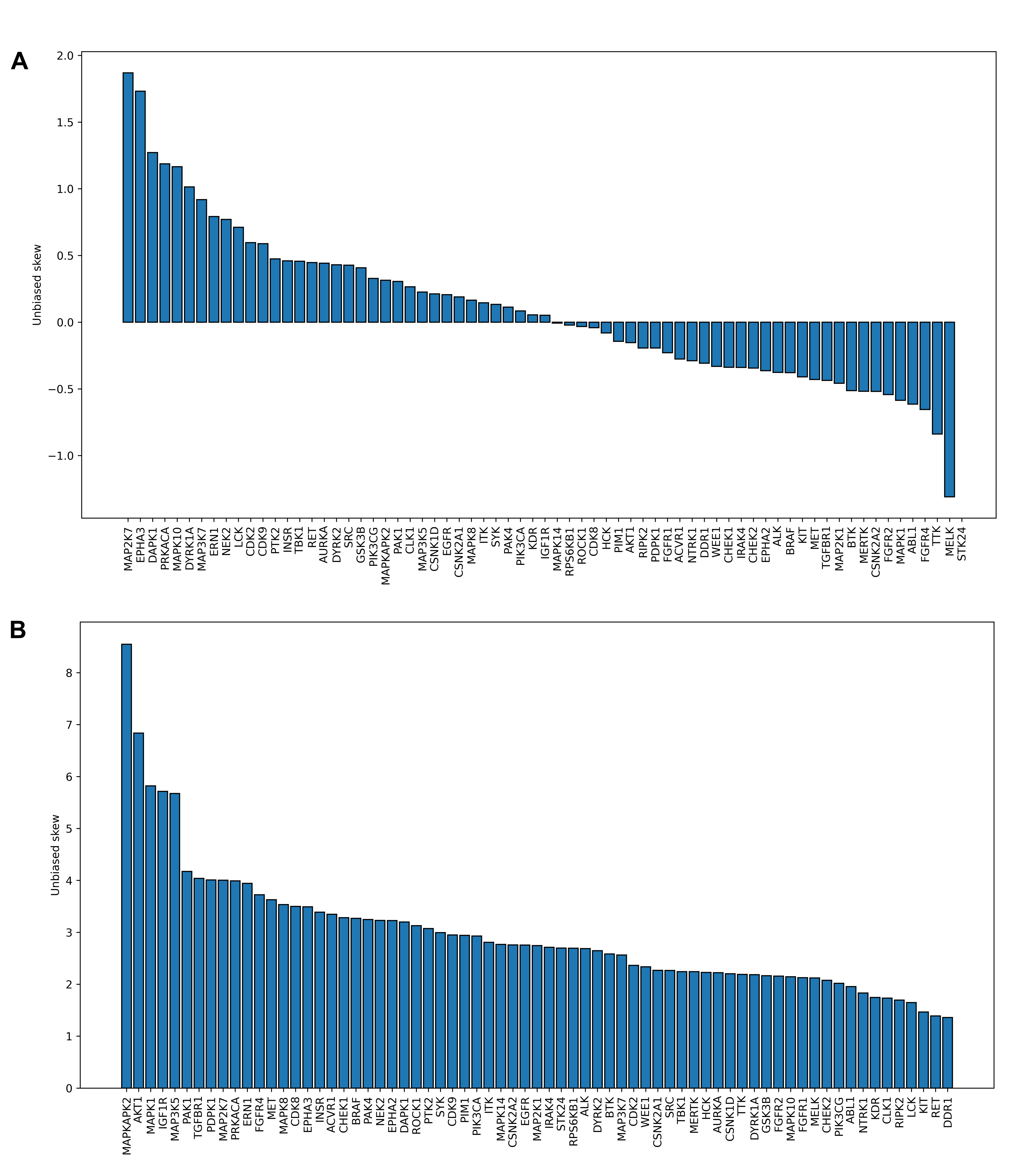

Supplement: S1 Fig — A) The sparse test set. B) The dense test set. The dense test set is skewed to the left. (TIF) [file pcbi.1011301.s001.tif]

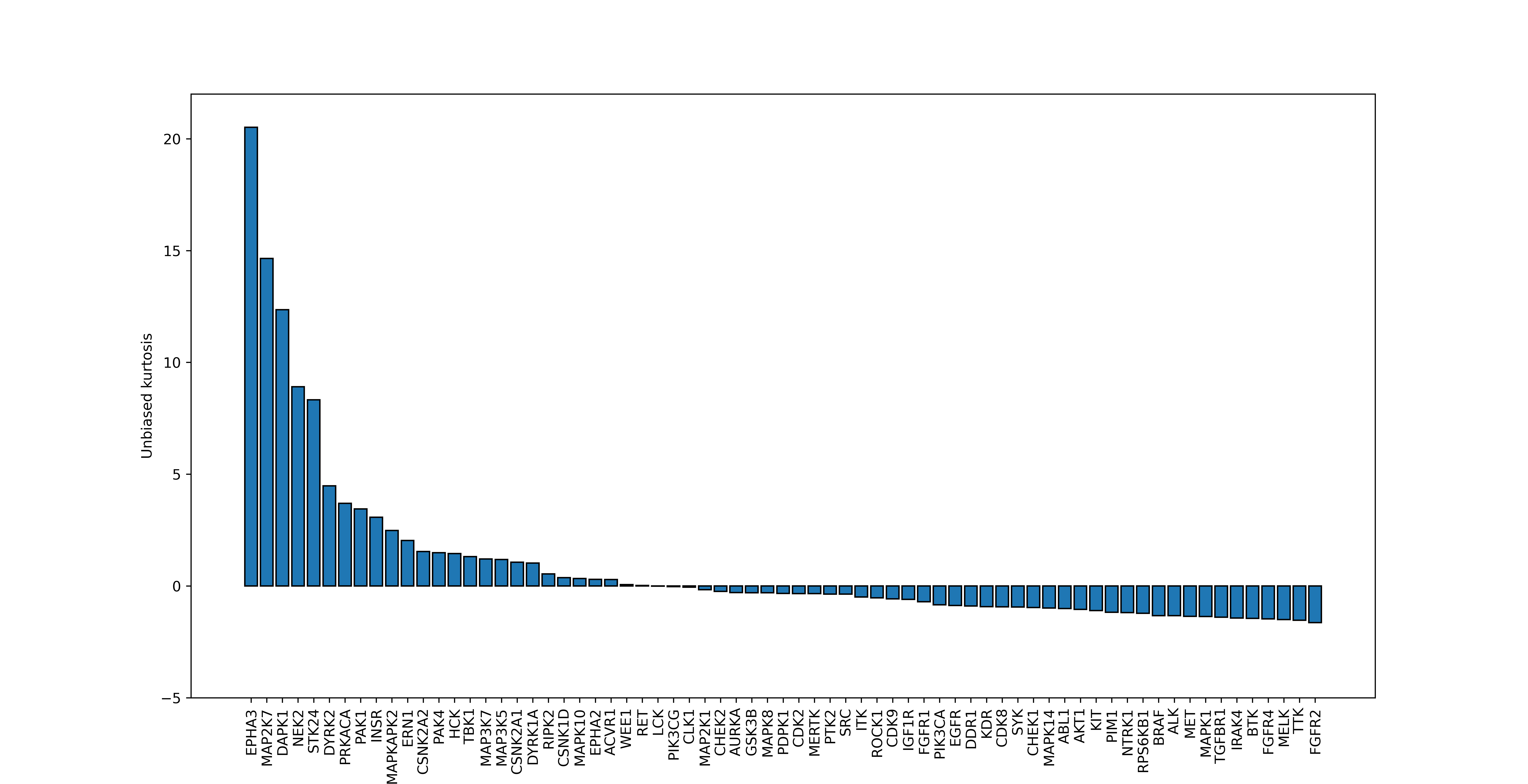

Supplement: S2 Fig — The plot was generated with the kurtosis function (using Fisher’s definition) of pandas 1.3.5. (TIF) [file pcbi.1011301.s002.tif]
