# Supplementary material for: Predicting the target landscape of kinase inhibitors using 3D convolutional neural networks
Source: PLoS Comput Biol. 2023 Sep 5;19(9):e1011301. doi: 10.1371/journal.pcbi.1011301 (PMC10508635; doi:10.1371/journal.pcbi.1011301)
Supplement: Attachment — Submitted filename: Revised Kanev et al.pdf [file pcbi.1011301.s013.pdf]

The resubmission of this paper has seen many commendable improvements over the last version, with many concerns at least partially addressed. Especially the reworking where now 1 CNN is at the core of the whole procedure makes this work much more intuitive, and appears to have helped the overall performance too! There are still some mainly textual concerns that should be addressed before publishing, some of which were already raised in the first review. This is a general point: quite a few of the (very minor) textual improvements addressed by all reviewers have been answered in the rebuttal as 'This has been corrected', yet are unchanged in the resubmitted manuscript. This warrants a thorough check.

Major points:

- As all reviewers in the first round have noted, no code or code availability statement was included in the first submission. This revision does not appear to include either again.
- Despite agreeing with the concern that personalised medicine is outside the scope of this paper, the very first 2 words of the abstract are still 'personalised therapies', and although the wording has changed, the allusion is still strong that this somehow would be a per-patient analysis. In practice, only the mechanism of action of a drug might be elucidated (which in itself in my view is a worthy goal).
- Figure 1C lacks a y-axis label, caption also unclear. What 'skewness' metric is plotted?
- It is unclear how the coloring in Fig 1E was assigned. The t-SNE embedding is solely based on chemical fingerprints, yet the text seems to suggest that the kinase information is somehow influencing the clustering. Rephrasing to for example 'some clusters of small molecules form that preferentially inhibit specific kinase families' would be closer to the underlying mechanics.
- The new setup of the CNN is conceptually clearer, but not yet explained well in the manuscript. The learning objective (recognising which specific kinase a structure belongs to) is not specified in either the manuscript, nor the methods. The size of the 3D FP is not detailed, nor is it specified anywhere which input was given for a specific fingerprint. One of the structures in KLIFS? Backpropagated from the ideal one-hot encoded output layer? **These are crucial details that allow readers to understand and reproduce the method, that are now missing.**
- Line 165: as mentioned before, Tanimoto 'score' is unclear. Please use similarity or distance to avoid confusion.

Minor textual/formatting points:

- Line 93: 'reveals that kinase have' – fix grammar
- Line 95: BRAF is depicted on the right of the skewness plot. "left of the distribution" -> Right
- Line 108: 'the t-SNE plot'
- Line 141: 'the' missing before sparse, comma dangling after a space
- Line 144: 71 should be updated to 69
- Line 171: decrease -> decreases (2x)
- Line 209: time -> times
- Line 486: Boxplots are not listed in order of Figure
- Line 487: panel D is captioned twice, second should be updated to E)
- Line 489 : packlage -> package
- Line 522 : suggestion to add 'true' cut-off information
- Line 539 : 71 chose kinases -> presumably now only 69 (did not count), also, chose<u>n</u> kinases
- SI Figure 1 lacks a y-axis label
- SI Figure 2 lacks a y-axis label
