# Supplementary material for: Predicting the target landscape of kinase inhibitors using 3D convolutional neural networks
Source: PLoS Comput Biol. 2023 Sep 5;19(9):e1011301. doi: 10.1371/journal.pcbi.1011301 (PMC10508635; doi:10.1371/journal.pcbi.1011301)
Supplement: Attachment — Submitted filename: Kanev et al Responses to reviewers comments.docx [file pcbi.1011301.s012.docx]

**General feedback to all reviewers:**

- Upon suggestion of a reviewer, the convolutional neural network part has now been revised to only one model. In its current form, a single 3D Convolutional Neural Network was trained to learn from the available kinase structures and generate the 3D FP fingerprints. The last layer of 3D CNN consists of 69 units, each representing a kinase. The flatten layer was used to generate the 3D FP.
- ChEMBL v28 was updated to v31 as v28 is already 2 years old.
- KIEO database has been removed for the following reason: tautomer issues were discovered in the annotation of some compounds. On their website, those issues were fixed, however we were not able to download what appears to be newer data (submitted a request without a reply). As precaution, we decided to remove the database. Also, the data is also not open source.

Reviewer's Responses to Questions

**Comments to the Authors:
Please note here if the review is uploaded as an attachment.**

Reviewer #1: In the paper entitled Predicting the target-landscape of kinase-inhibitors using 3D convolutional neural networks on densely covered chemogenomic data by Kanev et al. the authors devise and utilise a structurally derived fingerprint (FP) for the kinase domain of protein kinases, and compare the performance of their FP to other literature methods. They show performance on two literature derived datasets, one sparse, one dense, and conclude that their FP equals or slightly outperforms literature standards, depending on the dataset. The paper has a clear build-up, is well illustrated, but experimentally has serious shortcomings and lacks sufficient detail in the computational approach taken.

Major textual and conceptual problems:
**- The mention of personalised medicine and how this research supports this is poorly substantiated. Personalised medicine implies specific mutations or specific deregulations in a cancer, where this new FP might mostly give a clearer image in polypharmacology applications.**

***Response:*** *Thank you for this valuable feedback. Personalized medicine falls indeed outside the scope of this manuscript. We have adapted the text accordingly. In its current form, the manuscript does not suggest that the developed workflow is directly applicable to personalized therapies.*

**- From the introduction, it is unclear what the goal of this paper is. The fingerprint? The pipeline? The predicted affinity matrix? The introduction clearly lists a large amount of preceding papers that covered some (combination of) similar goals, yet the authors do not compare the performance of their models with any literature (CNN) model. The results shown, and the discussion thereof, does not extend at all beyond their own model and dataset. Literature model performance should at least be compared to show the merit of this new approach.**

***Response:*** *Thank you, this is very important comment. The primary goal of this paper is to introduce a new protein fingerprint that is learned using molecular structures and 3D convolutional neural networks. The learned fingerprints were then compared with other commonly used protein fingerprints like z-scales and ProtVec with random forest models. The sole purpose of random forest is to show the benefit of adding protein fingerprints (in addition to only inhibitor fingerprints) and to compare the different types of protein fingerprints. Random forest was chosen as it is one of the most commonly used algorithms, easy to implement and performs really well (especially given how easy it is to implement). The convolutional net was used only to extract structural molecular features that made the fingerprints. The net was not used to predict affinity and thus it does not make sense to compare it to other CNN models that predict affinities. The paper has been modified to better reflect the goal.*

**- From the text, it is unclear what the Random Forest models were tasked to predict. Talking about RMSE seems to imply that the RF model predicts -log[XC50]. This should be made way more obvious in the text. If this is indeed the case, correlation (R2) of predicted and observed values should be discussed and shown.**

***Response:*** *The random forest was indeed used to predict -log[XC50] using only inhibitor or inhibitor and protein fingerprints.R2 correlation score was added to the manuscript.*

**- Figure 4, and the supplementary tables, talk about true/false positives, but it is unclear what threshold value was set for a compound to qualify as ‘active’.**

***Response:*** *The threshold for active compound is 100 nM.*

**- The method of constructing a FP based on a CNN per protein is highly debatable. This strategy could be a major reason why many structures were needed per protein, as the resulting classification task was otherwise extremely challenging due to the imbalance of the underlying dataset. Variational Auto Encoders have previously been utilised for chemicals to generate an embedded latent space that is useable as FP for machine learning tasks (e.g. Winter et al.** [**https://pubs.rsc.org/en/content/articlehtml/2019/sc/c8sc04175j**](https://pubs.rsc.org/en/content/articlehtml/2019/sc/c8sc04175j)**), a more similar strategy might have worked here, also leading to a more general applicable FP that does not require the construction and training of a dedicated CNN per protein.**

***Response:*** *Thank you for this insightful feedback. We have now revised the CNN part – it is now a single model that learns from the protein kinase structures and generates the fingerprints. This is inspired by the variational autoencoder paper (and other autoencoders) as well as the fact that conv nets can be used to extract features. The model takes as input molecular structures (just like before) and predict to which kinase it belongs. The last output layer of the model has 69 units corresponding to each of the kinases. We made sure the imbalance in the data is (at least to an extend) corrected in the batches by balancing the provided structures by kinase. By doing this, the kinases are represented roughly equally on a batch level that is provided as input to CNN.*

**- The RF models for the dense dataset drop an incredible 0.48 in RMSE (1.67->1.19) by the addition of only 5 features in the z-scales whole sequence models. This dramatic effect of 5 features warrants more discussion.**

***Response:*** *Agree, we extended the discussion on this.*

**- The size of the dense dataset (155 compounds) is definitely on the small side, yet no comment is made on how this could impact model performance at all, instead the lack of performance is wholly blamed on the lack of target information (which apparently wasn’t a problem in the sparse dataset).**

***Response:*** *Thank you, we have now added discussion on size of test sets vs performance of the models.*

**- Practically, one could definitely argue that an RMSE of 0.82, 0.84, 0.87 or 0.92 is roughly equal in its reliability, showing that the addition of any protein data at all only very slightly improves model performance.**

***Response:*** *This is definitely true, most of the signal comes from the compound fingerprints. The addition of protein fingerprints does improve the performance, but not as much as compound fingerprints. Some of this could definitely be explained by the fact that the kinases are structurally very similar, while the compounds show much bigger variety. Also, most compounds are tested on a single kinase (or perhaps 2) and thus for those the multi-target data is simply missing. In other words, it is much easier to predict if a compound would bind to a kinase (any kinase) than to predict the exact kinase it would bind.*

**- It should be considered how the skewedness of the affinity values varies between the two datasets, and how this ties into model performance. Does the RF model just predict the median for that particular kinase in the dense dataset?**

***Response:*** *Skewness plots of the 2 test sets has been added to supplementary info. Regarding predicting the median value for a kinase, this has now been addressed in the text by analyzing what the RMSE would have been if a model always predicts the same value (namely the average potency of the training set compounds) and a model that always predicts the same value per target (namely the average potency of the training set compounds for that target).*

Minor textual points:
**- The cost of developing has increased 9-fold, 9 times implies 9 specific price hikes.**

*Corrected.*

**- Swapping Figure 1B and 1A helps to refer in the order mentioned in the text. Reason for having current Figure 1A is unclear, apart from mentioning that there are trends in conservation nothing is done with this information.**

***Response:*** *Good suggestion, A and B are swapped in the new version of figure 1. The part about Figure 1A is extended.*

**- In line 30, the reference 20 might be updated to the ’22 update, which also increases the 50 approved inhibitors to 68.**

***Response:*** *Thank you, modified the reference to “Robert Roskoski Jr, 2023: Properties of FDA-approved small molecule protein kinase inhibitors: A 2023 update”*

**- From the text, it is unclear whether the full protein sequence was used, or the kinase domain sequence.**

***Response:*** *The protein fingerprints (except 3D FP) use the 85 residues as defined by KLIFS for the Random Forest models. The description in the text has been improved.*

**- In line 133 the 1.4% disagrees with the 2.4% coverage mentioned earlier in the text.**

***Response:*** *This is now corrected.*

**- Figure 3 is said to show diversity of molecules (line 135), but only shows the number of inhibitors.**

***Response:*** *A cluster map is now used to better show the clusters of both inhibitors and kinases based on their activity data.*

**- Figure 3B shows ‘log activity’, where -log[inhibitor] is presumably meant.**

***Response:*** *The log activity is now -log[activity] where activity could be IC50, Kd or Ki or Potency.*

**- From the text (line 154) and Figure 4 it is unclear whether Tanimoto distance or similarity have been used. Based on the results, the similarity makes sense, but ‘score’ is not obvious.**

***Response:*** *Tanimoto similarity. This has now also been added to the text.*

**- No details at all have been given on the tSNE embedding generation. Hyperparameters, tSNE implementation etc. should be given to make the graph interpretable.**

***Response:*** *Details on how tSNE was generated have been added to the caption.*

Reviewer #2: The manuscript (ID: PCOMPBIOL-D-22-01124) entitled “Predicting the target-landscape of kinase-inhibitors using 3D convolutional neural networks on densely covered chemogenomic data” reports the development of a machine learning pipeline called 3DKINEssence, which is based on a novel type of structural fingerprints generated through 3D convolutional neural networks. The idea behind the study can be of high interest in modern drug discovery, especially in the contexts of kinase polypharmacology design and personalized medicine. However, the manuscript presents several issues that should be addressed.

**• A more thoroughly discussion on the concepts behind the generated 3D-CNN kinase fingerprints and on its main differences with respect to the other approaches encoding protein features should be provided. Perhaps, adding explanatory examples related to one or more compounds would help to better understand how the structural features of the proteins are perceived by the different approaches and the opportunities offered by the newly developed pipeline.**

***Response:*** *Thank you for your feedback. This is indeed very important aspect of the manuscript. We have extended the text to include more thoroughly discussion.*

**• The following sentences should be clarified to avoid potential misunderstandings: (i) page 5, lines 78-81; (ii) page 6, lines 105-10**

***Response:*** *Thank you, we have further clarified those sentences.*

**• The data activity skewness related to the kinases considered in the study was briefly discussed in the text and depicted in the right side of Figure 1A. A comment also on the kurtosis of the activities would help to better understand the data distribution across the different kinase datasets.**

***Response:*** *Thank you for spotting this. Kurtosis part has been added to the text.*

**• The full list of crystal structures used for the generation of the 3D FP should be provided as a supplementary file.**

***Response:*** *Thank you, a full list of the molecular structures has been added to supplementary info (Table 1).*

**• Figure 1 should be revised, as it presents a different number of panels with respect to those referenced in the text and in the corresponding caption. Moreover, the panels in Figure 1 should also be reorganized to make their reference in line with the text (for example, panel B now is referenced before panel A in the text). The type of molecular fingerprints employed for the generation of the tSNE plot should be clarified in the Figure 1 caption.**

***Response:*** *Thank you for bringing this into our attention. Figure 1 has been modified and panels were swapped so it matches the order of occurrence in the text. The type of molecular fingerprint is now included in the caption.*

**• Supplementary Table 1 should also provide information on the 9 groups to which the kinase proteins were assigned to, to better support the sentence at page 5, lines 77-78. Moreover, a reference to the Supplementary Table 2 is missing in the text.**

***Response:*** *The 3D Convolutional Neural Networks workflow has been modified to just one model (rather than a model per kinase): a single model is used to learn from all kinase structures and generate the 3D FP. This was suggested by a reviewer. This makes SI table 1 obsolete and we removed it. The kinase classification into groups is now supplementary table 1.*

**• The manuscript presents some typos and grammatical issues. I would suggest performing a careful revision of the text.**

***Response:*** *Thank you for spotting those. We have revised the manuscript to the best of our abilities.*

**• The bibliography is not exhaustive, albeit being already extensive. Few examples that would need to be referenced at their first occurrence in the text are: (i) “z-scales and ProtVec” at page 4, line 65; (ii) “Kieo database” at page 5, line 73; (iii) “Morgan fingerprints” at page 6, line 95; (iv) “KNIME analytical platform” at page 6 line 103; (v) “one-hot encoding” at page 7 line 119; (vi) “RDKit” at page 7, line 121; (vii) “UniProt” at page 19, line 508; (iix) “zScales CRAN package” at page 19, line 511; (ix) “PyTorch” at page 19, line 520; (x) “scikit-learn” at page 21, line 547.**

***Response:*** *The bibliography is now updated to include references of the mentioned packages.*

**• A more detailed description on the activity data filtering should be provided. This with particular regards to the type of assays considered for the activity data and how the presence of potential false positives records was managed.**

***Response:*** *This is indeed very important aspect and more detailed description has been included in the methods.*

Reviewer #3: The authors present a machine learning model using random forest (rf) regression for the prediction of activities of ligands for a set of 71 kinases. To this end, the binding pockets of kinases are encoded as 3D fingerprints. Those fingerprint together with ECFP4 fingeprints of ligands form the input of the rf model to predict potency of a ligand vs. the target encoded. The main novelty of their work stems from the construction of the 3D fingerprint using 3D convolutional neural network models. The manuscript is well written and structured making it easy to follow. This is aided by the clear and informative figures. However crucial information is missing from the manuscript that would prevent publication in its current form:

**Random forest models are built based on data extracted from ChEMBL and other sources. These methods are then tested on a sparse and dense data set. These data sets and their sources are not described in the manuscript. What are the activity annotations of the test sets (Kd, IC50, ...)? It also needs to be assured that this (or part of this) data is not also included in the training data. A meaningful evaluation of the results would only be possible if the independence of the test and training data sets can be assured.**

***Response:*** *Thank you for this feedback, it touches on a few very important points. Description of the databases has now included (extended) in the manuscript. Also, more detail has been provided in the methods section. The activity annotations in the test set are the same as in the training set, its either Kd, Ki or IC50 or inactive. A compound is considered inactive if Kd, Ki or IC50 >= 10,000 nM or it show < 5% activity or 95% inhibition (single point measurements). We indeed made sure that the test set is truly independent from the train set and the quality of the data is as high as possible (even though different assay have been used).*

**P3L25,P6L90: should refer to Figure 1C instead of 1B**

***Response:*** *This is corrected.*

**P6L94: should refer to Figure 1D instead of 1C**

***Response:*** *This is corrected.*

**P6L98: should refer to Figure 1E instead of 1D**

***Response:*** *This is corrected.*

**P6L109ff: Doing the math, the CNN models are trained and tested on highly unbalanced data. Assuming the minimum of 20 structures/kinase. The positive training set would consist on the order of 10 samples (50%) and the negative training set on the order of 70*10=700 samples from the other kinases. The reported performance would then be reported on the test set (I assume) of ca. 5 postive and 350 negative samples (25%). In this case precision and recall (and maybe F1-score as a combined measure) are indeed meaningfult, however accuracy is not. A model only predicting negative would already have an accuracy of ca. 0.985. As a combined score, either the balanced accuracy or a score like F1 could be reported instead.**

***Response:*** *Thank you, F1 and balanced accuracy has now been calculated as well. The convolutional neural network part has now been revised to only one model. A single 3D CNN was in this newer version trained on all structural data to learn and generate the 3D FP. However, the issue you address (unbalanced data) still remains and has been discussed in the text.*

**P19L25: The authors should provide a more details about the training and evaluation method, e.g., what are the absolute sizes of the training/test sets per target? (the supplemented contains the positive training set size, the sizes of the negatives sets could be included there per target); which kind of validation was done for hyperparameter optimization?; did the authors perform repeated trials?; and if so how were the final models for fingerprint generation chosen?**

***Response:*** *Thank you, the method section has been expanded to include more details on the training and evaluation methods as well as models and their parameters. We did not perform repeated trials (like cross-validation) and we also did not perform hyperparameter optimization. The pipeline was design in a way that keeps all the elements (with exception of the protein features) as rigid as possible in order to have more constructive results and discussion. If the individual models were trained with different parameters, the comparison becomes more challenging. Cross-validation would have actually been nice to have and show.*

**P6L111: Seems to refer to Figure 2 instead of Figure 3**

***Response:*** *This is corrected.*

**P7L139-140: "The root-mean-square-error ...": I feel the context of this statement is missing, to which test sets do the authors refer?**

***Response:*** *It refers to both test sets: sparse and dense. This sentence has been rewritten.*

**P7L139ff: To put the RSME results into persepective, the authors should provide the RSMEs for two baseline models:
1) A model that always predicts the same value, namely the average potency of tre training set compounds
2) A model that always predicts the same value per target, namely the average potency of the training set compounds for that target.**

***Response:*** *These are very good points, thank you. We have incorporated them in the discussion.*

**P8L153: How was the Tanimoto similarity determined? Was the most similar compound of the training set or some kind of average taken?**

***Response:*** *For each compound of the test set Tanimoto similarity was calculated by taking each compound of the train set and then the max value after the complete training set was exhausted. The compounds were prepared using morgan fingerprints with radius of 2 and 1024 bits length.*

**P9L168/Fig4B: How were True/False positives/negatives defined given that the models are regression models?**

***Response:*** *The threshold for active compound is 100 nM.*

**P17L459ff: Legend Figure1: The legend combines subfigures A and B as a single subfigure A. Legends/references in the text should be fixed. (The font of legend 1 does not match the font of the other figure legends.)**

***Response:*** *This has been corrected.*

**P19L512: chose -> chosen**

***Response:*** *This has been corrected.*

**P20Box 1: A lot of different activity types are mixed in the data preparation step: IC50, XC50, EC50, AC50, Ki, Kd, Potency the quantitative values will depend on assay conditions and are not comparable directly introducing a significant amount of error in the initial data. This issue needs to be addressed and/or discussed in some form in the manuscript. The authors should also investigate whether a model trained on a smaller training set of higher quality (restricting annotations to Ki/Kd (and IC50) would yield better results.**

***Response:*** *Thank you, this is really important comment. We have now used a newer version of ChEMBL v31 (compared to v28 previously) and were also stricter with the preparation to indeed ensure higher data quality. Currently, we use only Kd, Ki and IC50. Compounds having Kd/Ki/IC50 >= 10,000 nM are annotated as inactive. To expand on the inactive set, we also included compounds that show < 5% activity or 95% inhibition (single point measurements). Any other value for % activity or inhibition has been excluded, as it would not be possible to precisely determine its equivalent in -log[activity].*

**P21L554: B.W -> B.A.W.**

***Response:*** *This is corrected.*


**Figure 3B: Sparse data set: The figure is informative in the sense that it gives a good impression of the variance in the number of molecules per target. To this end, molecules were sorted according to their target from top to bottom along the x-axis. Given that the x-axis represents 8950 molecules, individual pixels shown cannot represent individual protein-ligand interactions. I would suggest, for the molecules within each target, these should be sorted in order of increasing (or decreasing) activity, so that the potency range for each target becomes visible.**

***Response:*** *Very good idea, figure 3B consists now of clustermaps that cluster both the compounds and the targets according to their activity profiles.*

**Have the authors made all data and (if applicable) computational code underlying the findings in their manuscript fully available?**
The [PLOS Data policy](https://eur04.safelinks.protection.outlook.com/?url=https%3A%2F%2Fjournals.plos.org%2Fploscompbiol%2Fs%2Fmaterials-and-software-sharing&data=05%7C01%7Ca.westerman%40amsterdamumc.nl%7C963094d845d54b97a66e08dab3397b3f%7C68dfab1a11bb4cc6beb528d756984fb6%7C0%7C0%7C638019356711173230%7CUnknown%7CTWFpbGZsb3d8eyJWIjoiMC4wLjAwMDAiLCJQIjoiV2luMzIiLCJBTiI6Ik1haWwiLCJXVCI6Mn0%3D%7C3000%7C%7C%7C&sdata=a9IDT4YP%2BZpr67wePsJSv%2BpbRR%2FRgTzYh5QB09V0SOc%3D&reserved=0) requires authors to make all data and code underlying the findings described in their manuscript fully available without restriction, with rare exception (please refer to the Data Availability Statement in the manuscript PDF file). The data and code should be provided as part of the manuscript or its supporting information, or deposited to a public repository. For example, in addition to summary statistics, the data points behind means, medians and variance measures should be available. If there are restrictions on publicly sharing data or code —e.g. participant privacy or use of data from a third party—those must be specified.

Reviewer #1: **No:** The supplementary tables detail median values, but separate values are not available. Code availability is not detailed in the paper.

Reviewer #2: None

Reviewer #3: **No:** No code has been provided. The activity annotations and structures of ligand of training and test set are not available.

PLOS authors have the option to publish the peer review history of their article ([what does this mean?](https://eur04.safelinks.protection.outlook.com/?url=https%3A%2F%2Fjournals.plos.org%2Fploscompbiol%2Fs%2Feditorial-and-peer-review-process%23loc-peer-review-history&data=05%7C01%7Ca.westerman%40amsterdamumc.nl%7C963094d845d54b97a66e08dab3397b3f%7C68dfab1a11bb4cc6beb528d756984fb6%7C0%7C0%7C638019356711173230%7CUnknown%7CTWFpbGZsb3d8eyJWIjoiMC4wLjAwMDAiLCJQIjoiV2luMzIiLCJBTiI6Ik1haWwiLCJXVCI6Mn0%3D%7C3000%7C%7C%7C&sdata=0cgW37M8nAXCyifUbiMTHQo84X1Jlp3GSG6w4LNl7GQ%3D&reserved=0)). If published, this will include your full peer review and any attached files.


If you choose “no”, your identity will remain anonymous but your review may still be made public.


**Do you want your identity to be public for this peer review?** For information about this choice, including consent withdrawal, please see our[Privacy Policy](https://eur04.safelinks.protection.outlook.com/?url=https%3A%2F%2Fwww.plos.org%2Fprivacy-policy&data=05%7C01%7Ca.westerman%40amsterdamumc.nl%7C963094d845d54b97a66e08dab3397b3f%7C68dfab1a11bb4cc6beb528d756984fb6%7C0%7C0%7C638019356711173230%7CUnknown%7CTWFpbGZsb3d8eyJWIjoiMC4wLjAwMDAiLCJQIjoiV2luMzIiLCJBTiI6Ik1haWwiLCJXVCI6Mn0%3D%7C3000%7C%7C%7C&sdata=uqYJUHI%2BqB1LolPSZvIW2jrgLBaPwkA86JT5nsFE%2BRA%3D&reserved=0).

Reviewer #1: No

Reviewer #2: No

Reviewer #3: **Yes:** Martin Vogt

Figure Files:

While revising your submission, please upload your figure files to the Preflight Analysis and Conversion Engine (PACE) digital diagnostic tool,[https://pacev2.apexcovantage.com](https://eur04.safelinks.protection.outlook.com/?url=https%3A%2F%2Fpacev2.apexcovantage.com%2F&data=05%7C01%7Ca.westerman%40amsterdamumc.nl%7C963094d845d54b97a66e08dab3397b3f%7C68dfab1a11bb4cc6beb528d756984fb6%7C0%7C0%7C638019356711173230%7CUnknown%7CTWFpbGZsb3d8eyJWIjoiMC4wLjAwMDAiLCJQIjoiV2luMzIiLCJBTiI6Ik1haWwiLCJXVCI6Mn0%3D%7C3000%7C%7C%7C&sdata=MPJWHKmExMZ25jod6nU%2FzVFcB2SiWhR7Y%2BY%2B9h1jvj4%3D&reserved=0). PACE helps ensure that figures meet PLOS requirements. To use PACE, you must first register as a user. Then, login and navigate to the UPLOAD tab, where you will find detailed instructions on how to use the tool. If you encounter any issues or have any questions when using PACE, please email us at [figures@plos.org](mailto:figures@plos.org).

Data Requirements:

Please note that, as a condition of publication, PLOS' data policy requires that you make available all data used to draw the conclusions outlined in your manuscript. Data must be deposited in an appropriate repository, included within the body of the manuscript, or uploaded as supporting information. This includes all numerical values that were used to generate graphs, histograms etc.. For an example in PLOS Biology see here: <http://www.plosbiology.org/article/info%3Adoi%2F10.1371%2Fjournal.pbio.1001908#s5>.

Reproducibility:

To enhance the reproducibility of your results, we recommend that you deposit your laboratory protocols in [protocols.io](http://protocols.io), where a protocol can be assigned its own identifier (DOI) such that it can be cited independently in the future. Additionally, PLOS ONE offers an option to publish peer-reviewed clinical study protocols. Read more information on sharing protocols at [https://plos.org/protocols?utm_medium=editorial-email&utm_source=authorletters&utm_campaign=protocols](https://eur04.safelinks.protection.outlook.com/?url=https%3A%2F%2Fplos.org%2Fprotocols%3Futm_medium%3Deditorial-email%26utm_source%3Dauthorletters%26utm_campaign%3Dprotocols&data=05%7C01%7Ca.westerman%40amsterdamumc.nl%7C963094d845d54b97a66e08dab3397b3f%7C68dfab1a11bb4cc6beb528d756984fb6%7C0%7C0%7C638019356711173230%7CUnknown%7CTWFpbGZsb3d8eyJWIjoiMC4wLjAwMDAiLCJQIjoiV2luMzIiLCJBTiI6Ik1haWwiLCJXVCI6Mn0%3D%7C3000%7C%7C%7C&sdata=cF4eY1MtAtYXoqBy4G3rgoeprUZKTfgisjotKDLPXQ4%3D&reserved=0)
