# Supplementary material for: Predicting the target landscape of kinase inhibitors using 3D convolutional neural networks
Source: PLoS Comput Biol. 2023 Sep 5;19(9):e1011301. doi: 10.1371/journal.pcbi.1011301 (PMC10508635; doi:10.1371/journal.pcbi.1011301)
Supplement: Attachment — Submitted filename: answers_to_reviewer_3.docx [file pcbi.1011301.s014.docx]

Reviewer #3: The authors have submitted a first major revision of their original manuscript
"Predicting the target-landscape of kinase-inhibitors using 3D convolutional neural
networks on densely covered chemogenomic data."

While the general approach has remained unchanged, the authors have changed the architecture of their 3D-CNN for the construction of protein 3D Kinases FPs. This modification significantly changes the generation of 3D-FPs, but it seems to be a meaningful modification over the original approach. Overall the manuscript has improved significantly, however some revisions are still required. While the written English is understandable, it could still improve from some editing, especially in the results section where it is on first reading often ambiguous whether given RMSE values refer to the performance of a model or the difference in performance between two models.

Points:

L20: "increased 9 times" -> "increased by a factor of 9"

L46: "random forest" -> random forests

L88: "large part" -> "a large part"

L91: "as result end in" -> "as a result ends in"

L92: "that kinase have " -> "kinases generally have"

L99: "than the sparse test set" -> "compared to the sparse set"

L100 -> "that most kinases are close to 0 " -> "it being close to 0 for most kinases"

***Response: all of the textual and formatting points were implemented. The overall quality of the text in the manuscript was improved with Grammarly (and us).***

L123/L546ff: According to manuscript of Fig. 2, the 3D-FP corresponds to the flatten layer. However, the size of the flatten layer and thus the 3D FP is not explicitly mentioned. This should be added here and/or in the main text (L123)

***Response: Thank you for this feedback, it’s very valuable. The methods section has been substantially expanded and it contains now detailed description and a table of the architecture of the model. It also contains information on how exactly the 3D FP were generated (including their size). The code responsible for training and evaluating CNN (including the generation of 3D FP) has also been uploaded to GitHub.***

L140ff/L572ff: The manuscript is still a bit unclear what training and test sets where chosen and how training was done. Were different models trained separately for the dense and the sparse data set? Was part of the data used for training and another for testing or was data from one source used for training and then tested on data from another source. The machine learning workflow should be extended to include this information in the methods and/or L140
***Response: Thank you for this very valuable feedback. The methods section answers now how the test sets were generated. The data from ChEMBL and the publication of Serge Christmann-Franck et al 2016 (PMID: 27482722) was merged into one, then split into train and test sets (once for the sparse set and once for the dense set). The different models were trained separately for the dense and sparse sets indeed. The 2 sets (i.e., dense and sparse) can be seen as 2 independent workflows that use the same merged data from ChEMBL and PMID: 27482722 (but different splits of train and test sets). All scripts are uploaded to GitHub:*** [***https://github.com/bartwesterman/Kanev_et_al_2023***](https://github.com/bartwesterman/Kanev_et_al_2023)***. Links to a few large files need to be added, the rest of the code (and the input and output files) is there. The authors will improve the description in the coming days.***

L180: The threshold potency values for positive and negative predictions should be explicitly stated in the manuscript and not only the reviewer responses.

***Response: Thank you, the*** ***threshold values were added to the text.***

Original points by the reviewer that were not/inadequately addressed:

"""
P6L109ff: Doing the math, the CNN models are trained and tested on highly unbalanced data. Assuming the minimum of 20 structures/kinase. The positive training set would consist on the order of 10 samples (50%) and the negative training set on the order of 70*10=700 samples from the other kinases. The reported performance would then be reported on the test set (I assume) of ca. 5 positive and 350 negative samples (25%). In this case precision and recall (and maybe F1-score as a combined measure) are indeed meaningfult, however accuracy is not. A model only predicting negative would already have an accuracy of ca. 0.985. As a combined score, either the balanced accuracy or a score like F1 could be reported instead.
**Response: Thank you, F1 and balanced accuracy has now been calculated as well. The convolutional neural network part has now been revised to only one model. A single 3D CNN was in this newer version trained on all structural data to learn and generate the 3D FP. However, the issue you address (unbalanced data) still remains and has been discussed in the text.**
"""
Comment: I could not find the discussion but the point should be moot due to the changed architecture of the CNN. However, The SI contains balanced accuracy and F1 scores for the RF models, which is appreciated.

"""
P7L139ff: To put the RSME results into persepective, the authors should provide the RSMEs for two
baseline models:
1) A model that always predicts the same value, namely the average potency of tre training set
compounds
2) A model that always predicts the same value per target, namely the average potency of the training set
compounds for that target.
**Response: These are very good points, thank you. We have incorporated them in the discussion.**
"""

Comment: The values are report in the supplemental information, but I could not find a discussion of them in the manuscript.

***Response: Thank you, the discussion has been added to the results section of the text.***
